# Supplementary material for: Crystal-by-Crystal Assembly in Two Types of Periodically Banded Aggregates of Poly(p-Dioxanone)
Source: Polymers (Basel). 2023 Jan 11;15(2):393. doi: 10.3390/polym15020393 (PMC9866735; doi:10.3390/polym15020393)
Supplement: Supplementary file 1 [file polymers-15-00393-s001.zip › polymers-2129169-supplementary.pdf]

Supporting Information

Crystal-by-Crystal Assembly in Two Types of Periodically Banded Aggregates of Poly(p-dioxanone)

Kuan-Ying Huang[1], Yu-Zhe Huang[1], Li-Ting Lee[2] and Eamor M. Woo[1,]

1. Department of Chemical Engineering, National Cheng Kung University
No. 1, University Road, Tainan, 701-01, Taiwan
2. Department of Materials Science and Engineering, Feng Chia University, Taichung, 407-24, Taiwan.
Correspondence: emwoo@mail.ncku.edu.tw; Tel.: +886 6 275-7575 x 62670.

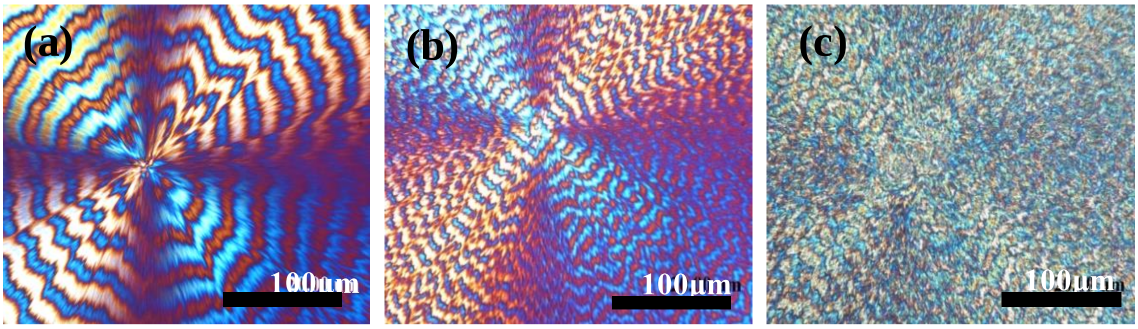

Figure S1. POM graphs of PPDO crystallized at higher Tc: (a) 80 oC, (b) 85, (c) 90 oC, showing increasingly corrupted bands with lesser regularity. Two types of bands disappear at high Tc.

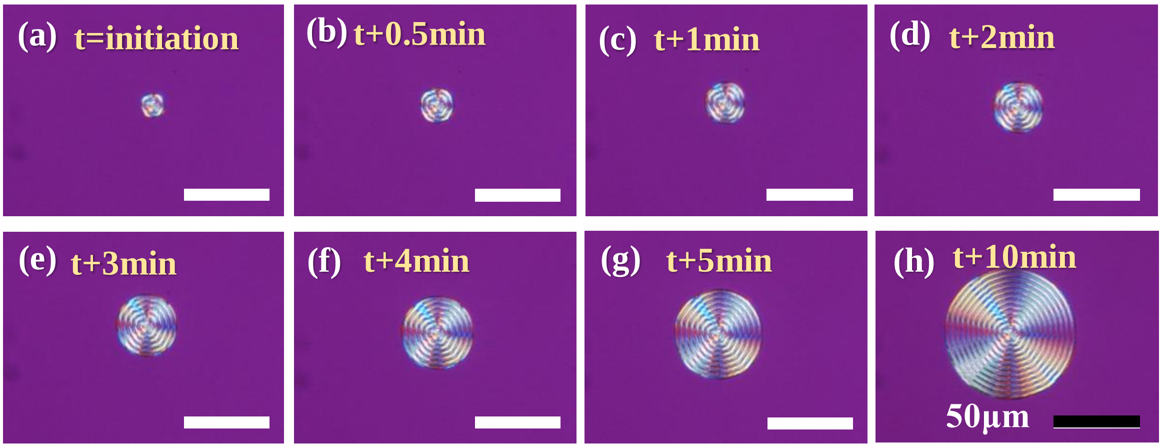

Figure S2. (a)-(h) In-situ POM graphs of PPDO captured at Tc = 76oC at different times (0.5 – 10 min) of crystallization by quenching from Tmax = 150oC. (max. melt temp. from which specimens were rapidly quenched to isothermal Tc).

T_c	Type-p no. %	Type-n no. %
70	75	25
72	72.7	27.3
74	65.4	34.6
76	96	4
78	75	25

Table S1. Variation of number fractions of Type-p and Type-n spherulites of PPDO at increasing T_c = 70 °C, 72 °C, 74 °C, 76 °C, and 78 °C. (Type-n no. % = 100% - (Type-p no. %).

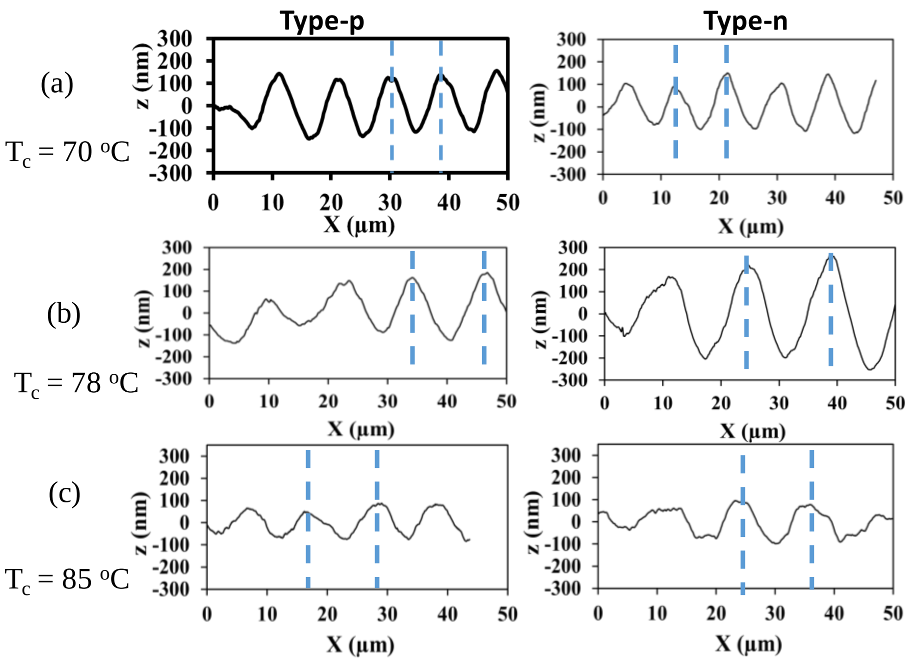

Figure S3. AFM height profiles of Type-p and Type-n of PPDO crystallized at various T_c: (a) 70 °C, (b) 78 °C, (c) 85 °C.
